# Towards Preventative Psychiatry: Concurrent and Longitudinal Predictors of Postnatal Maternal-Infant Bonding

**DOI:** 10.1007/s10578-022-01365-0

**Published:** 2022-05-26

**Authors:** Frances L. Doyle, Sophie J. Dickson, Valsamma Eapen, Paul J. Frick, Eva R. Kimonis, David J. Hawes, Caroline Moul, Jenny L. Richmond, Divya Mehta, Mark R. Dadds

**Affiliations:** 1 School of Psychology, Faculty of Science, University of Sydney, 2006 Sydney, NSW Australia; 2 School of Psychology; MARCS Institute for Brain Behaviour and Development; Transforming early Education And Child Health Research Centre, Translational Health Research Institute, Western Sydney University, 2750 Penrith, NSW Australia; 3 Faculty of Medicine, Health, and Human Sciences, Macquarie University, 2109 Sydney, NSW Australia; 4 School of Psychiatry, Faculty of Medicine, University of New South Wales, 2052 Kensington, NSW Australia; 5 Department of Psychology, Louisiana State University, 70803 Baton Rouge, LA USA; 6 School of Psychology, University of New South Wales, 2052 Kensington, NSW Australia; 7 Centre for Genomics and Personalised Health, Faculty of Health, Institute of Health and Biomedical Innovation, Queensland University of Technology (QUT), Kelvin Grove, 4059 Brisbane, Queensland Australia

**Keywords:** Maternal bonding, Pregnancy, Postnatal, Women’s mental health, Postpartum Bonding Questionnaire

## Abstract

Maternal-infant bonding is important for children’s positive development. Poor maternal-infant bonding is a risk factor for negative mother and infant outcomes. Although researchers have examined individual predictors of maternal-infant bonding, studies typically do not examine several concurrent and longitudinal predictors within the same model. This study aimed to evaluate the unique and combined predictive power of cross-sectional and longitudinal predictors of maternal-infant bonding. Participants were 372 pregnant women recruited from an Australian hospital. Data were collected from mothers at antenatal appointments (T0), following their child’s birth (T1), and at a laboratory assessment when their child was 5-11-months-old (T2). Poorer bonding at T2 was predicted at T0 by younger maternal age, higher education, and higher antenatal depressive symptoms. Poorer bonding at T2 was predicted at T1 by younger maternal age, higher education, and higher postnatal depressive symptoms. Poorer bonding at T2 was predicted at T2 by younger maternal age, higher education, higher postnatal depression symptoms, higher concurrent perceived social support, and more difficult infant temperament, when controlling for child age at T2. To promote positive maternal-infant bonding, global and targeted interventions in the perinatal period may benefit from targeting maternal psychopathology, perceived lack of social support, and coping with difficult infant temperament.

A strong mother-infant bond is considered crucial for promoting positive parenting behaviours and optimal child psychosocial development [1, 2]. Maternal-infant bonding (MIB) refers to a mother’s perceived emotional connectedness to her infant [2, 3]. MIB often begins to develop antenatally, grows significantly through the first year of life, and continues to develop throughout the lifespan [4–6]. Impaired bonding, however, manifests in a range of behaviours, including irritability and hostility toward the infant, absence of positive feelings, as well as dismissal of the infant [1]. Impaired MIB is a risk factor for a range of negative infant and maternal outcomes; and, at the extremes, may manifest as severe clinical syndromes – or disorders – where there is emotional rejection of the infant and/or pathological anger toward the infant [1, 7–9]. It has been estimated that these severe clinical syndromes occur in about 1% of births in the general population [9], yet causes are still not well-known [7]. Since even sub-clinical levels of emotional rejection of the infant may be related to negative infant and maternal outcomes, warranting intervention or prevention efforts, it may be useful to understand what factors predict poorer MIB in the general population, particularly when these factors are considered in the same statistical model; that is, to determine which variables are uniquely predictive above and beyond other known variables in the model. Further, few studies provide a longitudinal analysis and thus little is known about when variables of interest may be more strongly related to poor MIB.

## Mother-Related Variables

The detrimental effects of postnatal depression on poor MIB have been well documented cross-sectionally [5, 10], and longitudinally [11–13]. Some mothers experiencing postnatal depression may not be sensitive to infant cues, or may be withdrawn, intrusive, apathetic or angry, which can sometimes result in infant rejection [14, 15]. While postnatal depression is understood to be a major contributor to poor bonding, not all mothers with postnatal depression experience impaired bonding nor do all mothers with impaired MIB have postnatal depression. Additionally, many other predictors of poor MIB appear to be interconnected with postnatal depression, such that they may influence, or be influenced by postnatal depression. Thus, it is yet to be discerned whether other predictors play a role above and beyond their interrelationship with postnatal depression.

Researchers examining poor MIB have tended to pay greater attention to postnatal depression than postnatal anxiety. To our knowledge, ten studies have investigated maternal anxiety and poor MIB in the early postpartum period [6, 16–24]. Nine of these studies reported that maternal anxiety was associated with poorer bonding [16–24], but five of these reported associations did not hold their significance after controlling for postnatal depression [17, 19, 22–24]. One study reported that maternal anxiety was associated with better bonding scores [6]. Thus, the evidence for a relationship between maternal anxiety and poor MIB is sparse and mixed. The current study will be able to shed light on the relationship between postnatal anxiety and poor MIB, when controlling for postnatal depression.

Three further factors that may be related to poor MIB are antenatal attitudes towards the pregnancy, social support, and maternal history of maltreatment. Antenatal attitudes towards the pregnancy have been linked to poor MIB across clinical observations of severe clinical emotional rejection of the infant, yet have not been examined in cohort studies [7, 8]. Brockington [7] suggested that three groups should be assessed – namely: planned pregnancies, unplanned but accepted pregnancies, and unwanted pregnancies – with greater risk for poor MIB likely occurring in those with unwanted pregnancies. Additionally, poor satisfaction with social support systems during pregnancy has been associated with lower quality MIB [25, 26]. Further, maternal history of maltreatment has been associated with impaired MIB via maladaptive parenting practices and psychopathology in adulthood [11, 27].

### Birth and Breastfeeding Experiences

Dissatisfaction with childbirth has been purported to disrupt some mothers’ abilities to engage in responsive and sensitive caregiving behaviours that are needed to facilitate positive MIB [28]. Although dissatisfaction with the childbirth experience (particularly due to mother-infant physical separations) was initially thought to be central to bonding impairments [2, 29], findings from past studies have been mixed and further research is therefore warranted [3, 28].

The typical preterm infant also has characteristics that are known to make bonding difficult, including responsiveness problems, irritability, and being harder to soothe [30, 31]. These infant characteristics have been associated with reduced maternal sensitivity, emotional detachment, and an elevated risk for child abuse and neglect [32]. Yet some studies have reported no differences in feelings of bonding, and even increased maternal investment in mothers of preterm infants compared to mothers of full-term infants [33, 34]. As antenatal depression has been identified as a risk factor for preterm birth [35], it is possible that the mixed findings reflect variations in maternal mental health, as none of the aforementioned studies controlled for maternal depression antenatally or postnatally.

While the positive effects of breastfeeding on poor MIB are often promoted by public health organisations and are often assumed in the literature, findings in this area are inconsistent. Some studies have found no relationship between breastfeeding status and bonding quality [36–38]; whereas others have found that breastfeeding mothers show more affectionate responses during breastfeeding [39], lower maternal negative affect [40], and an overall higher MIB than non-breastfeeding mothers [41–43]. It is evident, however, that not all studies control for depression symptoms. Depression has been shown to have a bidirectional effect on breastfeeding status (e.g., [44, 45]); with depressed mothers more likely to stop breastfeeding earlier than non-depressed mothers, and earlier cessation of breastfeeding has also been associated with depression. Thus, further investigation of the association between breastfeeding status and poor MIB is warranted, particularly controlling for depression symptoms. In line with previous research (e.g., [38]), breastfeeding status in this study will be a dichotomous variable that indicates whether or not the mother was breastfeeding at the time of the assessment.

### Child Temperament

Temperament is defined as biologically based individual differences in reactivity and self-regulation (Gartstein & Rothbart, 2003; Rothbart, 1981; Rothbart et al., 2001). In this study, the term “difficult infant temperament” is used to describe infant temperaments that are characterized by high negative affect (Rothbart & Gartstein, 2000). Difficult infant temperament is known to impact bonding-related factors like negative maternal responses, reduced maternal responsiveness, parenting stress, and mutual rejection between the mother and infant [16, 46–48] but has not often been studied in relation to MIB itself. While difficult infant temperament has been identified as a predictor of postnatal depression [49], it is also understood that depressed mothers are more likely to perceive bonding to their infant more negatively [50]. Nolvi and colleagues [16] found significant unique relationships between high mother-reported infant negative emotionality and low mother-reported infant positive emotionality with poor MIB, over and above maternal depression and anxiety. These findings highlight the importance of considering child and mother characteristics together within the same model.

### Maternal Characteristics

Maternal characteristics, such as age and education, have been linked to poor MIB. While some studies find that younger women report higher quality bonding with their infants than older women [4, 38], other studies find no association between bonding and age [6, 51, 52]. In regard to education levels, findings are mixed. Some studies have shown that MIB does not differ by maternal education [6, 13, 21]; whereas, others have shown that women with lower levels of education have reported better bonding with their infant compared to women with higher levels of education [4, 5, 38]. Studies that link education with poor MIB have purported that they believe this to be due to those with more education being more inclined to answer more honestly about their relationship with their infant [4, 5, 38].

### The Current Study

The aim of the current study was to examine whether postnatal depression, postnatal anxiety, antenatal attitudes towards the pregnancy, perceived social support, maternal history of maltreatment, dissatisfaction with childbirth, breastfeeding, difficult infant temperament, gestation, maternal age and maternal education were predictors of poor MIB in a general population sample. To date, no prior studies have empirically examined how these factors operate in relationship to poor MIB when considered together and, as noted above, this is particularly important in relation to maternal depression. Thus, this is the first study to prospectively investigate a comprehensive range of factors in relation to poor MIB. Based on previous research, it was hypothesised that all factors would be significantly bivariately correlated with poor MIB. Given previous research, it was expected that maternal postnatal depression would be the strongest predictor for poor MIB. While some previous studies control for maternal postnatal depression, not all do. It was therefore hypothesised that several of these variables would no longer correlate with poor MIB when examined in a model with other predictors, particularly postnatal depression.

## Participants, Ethics, and Methods

### Participants

The current paper reports on data from the ‘Watch Me Grow for REAL’ study [53], which follows 788 mother-child dyads prospectively from mother’s antenatal appointments until children turn 3-years-old. Mothers were recruited from the postnatal ward of an Australian hospital between October 2017 and December 2018. The inclusion criteria for the broader ‘Watch Me Grow for REAL’ study were having a child born at Liverpool hospital in Sydney, Australia during the recruitment period, maternal intention to remain in Sydney for four years, and maternal functional English (or a family member willing, and able, to translate throughout the project). The exclusion criteria for the broader ‘Watch Me Grow for REAL’ study was if the child was known to have congenital conditions at birth. Mothers were also excluded from the study if nursing staff reported to the research team that the mother will not be taking her child home from the hospital (e.g., infant death, removal of the child from maternal care at the hospital by child services).

Participants in this study were a sub-sample of mothers (*n* = 372) who completed the Postpartum Bonding Questionnaire (PBQ) at T2 [54, 55]. When both twins were recorded in the sample, we excluded the youngest twin (*n* = 11) to ensure statistical independence between mother reports. Mothers’ age at birth ranged from 18.28 to 44.54 years (*M* = 31.19, *SD* = 5.06). Mothers in the sample gave birth to 200 boys (53.8%) and 172 girls (46.2%). Mothers were of Caucasian (*n* = 109, 29.3%), Middle-Eastern (*n* = 77, 20.7%), South Asian (*n* = 62, 16.7%), South-East Asian (*n* = 57, 15.3%), African/African American (*n* = 17, 4.6%), Polynesian/Melanesian (*n* = 18, 4.8%), Hispanic/Latino (*n* = 14, 3.8%), East Asian (*n* = 12, 3.2%), and Aboriginal/Torres Strait Islander (*n* = 4, 1.1%) descent, and two mothers had missing ethnicity data (0.5%). At the child’s birth, 294 (79.0%) mothers were married, 66 (17.7%) were in a relationship, 9 (2.4%) were single, and three mothers had missing relationship status data (0.8%). Mothers were reimbursed for their participation in the T2 assessment with a $40 gift voucher.

To assess for selective attrition bias, comparisons were conducted between this sub-sample and the 396 mothers who did not complete the PBQ (i.e. did not attend the T2 assessment, or who attended T2 but did not have time to complete this questionnaire). Mothers of twins were counted once across measures. There were no significant differences on child gender, child gestation, maternal experience of child abuse, antenatal depression symptomology, childbirth satisfaction, or breastfeeding status at birth (all *p*s > 0.05). However, mothers who completed the PBQ were significantly older (*M*_*PBQ*_ = 31.19 years *vs. M*_*No PBQ*_ = 29.84 years, *F*(1, 767) = 12.00, *p* = .001), more educated (χ2 (*df* = 5, *N* = 757) = 36.14, *p* < .001), perceived less social support antenatally (χ2 (*df* = 1, *N* = 757) = 6.02, *p* = .014) and at birth (χ2 (*df* = 1, *N* = 762) = 6.04, *p* = .014), and more depressed following their child’s birth (*M*_*PBQ*_ = 4.24 *vs. M*_*No PBQ*_ = 3.54, *F*(1, 650) = 6.37, *p* = .012) than those who did not complete the PBQ at T2.

## Ethics

This study involved human participants. It was reviewed and approved by The University of Sydney Human Research Ethics Committee (Project Number 2017/644), and the South Western Sydney Local Health District Human Research Ethics Committee (Local Project No HE17/115). Written informed consent was obtained from mothers for their participation and mothers provided written informed consent for the participation of their child.

## Measures and Procedure

Data from antenatal records (when women were approximately 20 weeks gestation; T0), birth records and self-reports (T1), and a laboratory assessment when children were aged between 5- and 11-months (T2) are reported in the present study.

**Antenatal data.** At the time of recruitment, mothers gave consent for the researchers to access data from an antenatal interview [56] conducted with a midwife at approximately 20 weeks gestation. Variables extracted from this interview include maternal experience of child abuse (*none* *reported,* *child abuse reported*), lifetime history of anxiety and/or depression (*no, yes*), whether mothers’ perceived they will have good social support following birth (*perceived not enough support following birth, perceived they would have good social support following birth*). Antenatal attitudes towards pregnancy were captured using four categories (i.e. *planned and happy; planned and unhappy; unplanned and happy; unplanned and unhappy*). As no mothers endorsed the category ‘*planned and unhappy*’ and this category was not indicated in previous theoretical papers [7], only three categories were carried forward (i.e. *planned and happy, unplanned and happy, unplanned and unhappy*).

Mothers responded on the Edinburgh Postnatal Depression scale [EPDS; 57, 58]. The EPDS is a ten-item scale that has been validated in numerous trials and languages for the detection of postnatal depression (e.g., [59, 60]). The total score from the EPDS indicated mothers’ depression symptomatology during the past week, with higher scores indicating greater antenatal depressive symptoms and a score of 13 or more indicating antenatal depressive symptoms above the clinical threshold.

Demographic items were also self-reported on our Newborn Baseline Questionnaire (NBQ; [61]) at the time of recruitment (i.e. usually within one week of the child’s birth). These variables included mothers’ ethnicity, highest level of education completed (*primary school, Year 10 or equivalent, Year 12 or equivalent, TAFE qualification or trade apprenticeship, undergraduate university degree, postgraduate university degree*), and number of years living in Australia (*0–5 years, 6–10 years, 11–15 years, 16–20 years, 21 + years*).

**Birth-related data.** Routinely collected birth data (recorded by midwives and doctors) and postnatal Edinburgh Postnatal Depression scale [EPDS; 57, 58] scores (recorded by Community Health Nurses within the month following birth when conducting home visits) were extracted from mothers’ electronic medical records. Birth-related data included mother’s age at the birth of her child, birth type (*vaginal unassisted birth, vaginal assisted birth, elective Caesarean section, emergency Caesarean section)*, and gestational age. Mothers also self-reported the following constructs on the NBQ: satisfaction with childbirth (see details below), current breastfeeding status following birth (*not breastfeeding, currently breastfeeding*), perceived social support (*perceived not enough support, perceived good support*), and overall relationship satisfaction (see details below).

Mothers responded to the 7-item Satisfaction With Childbirth scale [SWCh; 62] on a 7-point Likert scale ranging from 1, *strongly disagree*, to 7, *strongly agree*. Scores on the SWCh reflect mothers’ satisfaction with the childbirth experience, with higher scores indicating greater satisfaction. The current study demonstrated good internal consistency (Cronbach’s alpha = 0.83) in line with previous studies [62, 63].

Mothers were asked to respond to the Overall Relationship Satisfaction item from the Dyadic Adjustment scale [64]. Mothers reported, on a 7-point scale, ranging from 0, *extremely unhappy*, to 6, *perfect*, how happy they were with their relationship with the baby’s biological father.

**T2 data from the laboratory assessment.** Mothers self-reported the following constructs at a laboratory visit when their child was aged between 5- and 11-months-old: perceived social support perceived social support (*perceived not enough support, perceived good support*), current breastfeeding status (*not breastfeeding, currently breastfeeding*), and overall relationship satisfaction with the baby’s biological father.

Mothers also reported on their own mental health during the past week using the 21-item Depression Anxiety Stress Scales (DASS21) [65]. The DASS21 has been validated in both research and clinical settings [66, 67]. Mothers rated on a four-point scale, ranging from 0, *did not apply to me at all*, to 3, *applied to me very much, or most of the time*. Higher scores indicate more frequent symptomology. In this study, only the depression subscale (Cronbach’s alpha = 0.79) and the anxiety subscale (Cronbach’s alpha = 0.71) are reported.

Further, mothers reported on their child’s temperament using the Infant Behavior Questionnaire (IBQ) Revised - Very Short Form [68]. Mothers reported, on a 7-point scale, ranging from 1, *never*, to 7, *always*, the frequency with which their child enacted specific behaviours over the past 7 days. Mothers were also given the option to select “*does not apply*”, and if selected, that item is excluded from the scale score. The validity of its’ predecessors – IBQ and IBQ Short form – has been extensively documented, with research demonstrating their convergent validity with observational measures [69]. In this study, only the negative affect subscale is examined with higher scores indicating greater difficult infant temperament. Cronbach’s alpha was 0.78 in the current study, which was consistent with earlier use of ‘Very short’ form of the IBQ- R (e.g., [70]).

**Maternal-infant bonding.** At T2, mothers completed the 23-items from the PBQ [54, 55]. Numerous studies have found the PBQ to be both a valid and reliable tool for identifying women at risk of impaired mother-infant bonding [5, 55, 71]. The questionnaire contains items addressing the mother’s feelings towards her infant such as “*My baby irritates me*”. The response categories range from 0, *never*, to 5, *always* on a six-point scale (Cronbach’s alpha = 0.87), with higher scores indicating poorer MIB.

## Analytic Plan

Bivariate correlations were examined. Three regression models were run with poor MIB as the outcome variable. First, a regression model was run with the antenatal (T0) variables predicting poor MIB. In Step 1, were the maternal characteristics (i.e. mothers’ age and education). In Step 2, was antenatal depression. In Step 3, were the antenatal mother-related variables (i.e. maternal experiences of child abuse, antenatal perceived social support, and attitudes towards pregnancy).

Second, a regression model was run with the birth-related (T1) variables predicting poor MIB. In Step 1, were the maternal characteristics (i.e. mothers’ age and education). In Step 2, was postnatal depression. In Step 3, were the T1 mother-related variables (i.e. maternal experiences of child abuse, T1 perceived social support, and attitudes towards pregnancy). In Step 4, were the birth and breastfeeding variables (i.e. satisfaction with childbirth, breastfeeding status, and gestation).

Third, a regression model was run with poor MIB as the outcome variable, and all T0, T1, and T2 variables predicting MIB. In Step 1, were the maternal characteristics (i.e. mothers’ age and education). In Step 2, were the depression variables at T0, T1, and T2. In Step 3 were the T1 mother-related variables (i.e. maternal experiences of child abuse, T0-T2 perceived social support, attitudes towards pregnancy, T2 anxiety). In Step 4, were the birth and breastfeeding variables (i.e. satisfaction with childbirth, breastfeeding status, and gestation). In Step 5, were the child characteristics (i.e. difficult infant temperament).

## Results

### Description of the Sample

Descriptions of the sample from the time of recruitment, the time of birth, and the time of the T2 assessment can be found in Tables 1 and 2, and 3, respectively.

**Table 1:** Description of the Sample from Antenatal Data

	*n* (%)
**Maternal Experience of Child Abuse**	
None reported Child abuse reported Missing	323 (86.8) 39 (10.5) 10 (2.7)
**Lifetime History of Anxiety and/or Depression**	
No Yes Missing	276 (74.2) 84 (22.6) 12 (3.2)
**Perceived Social Support**	
Not enough support Good support Missing	20 (5.4) 348 (93.5) 4 (1.1)
**Antenatal Attitudes Towards Pregnancy**	
Planned and happy Unplanned and happy Unplanned and unhappy Missing	236 (63.4) 128 (34.4) 2 (0.5) 6 (1.6%)
**Depressive Symptoms**	
Depressive Symptoms Score (*n* = 357) Missing Scored Above Clinical Threshold	*M* = 5.22, *SD* = 4.18, 15 23 (6.4)
**Education**	
Primary School Year 10 or Equivalent Year 12 or Equivalent TAFE qualification or Trade Apprenticeship Undergraduate University Degree Postgraduate university Degree Missing	5 (1.3) 27 (7.0) 53 (14.2) 83 (22.3) 131 (35.2) 72 (19.4) 2 (0.5)
**Number of years Living in Australia**	
0–5 Years 6–10 Years 11–15 Years 16–20 Years 21 + Years Missing	72 (19.4) 63 (16.9) 30 (8.2) 27 (7.3) 175 (47.0) 5 (1.3)

**Table 2:** Description of the Sample from Data Collected at Birth

	*n* (%)
**Depressive Symptoms**	
Depressive Symptoms Score (*n* = 343) Scored above Clinical Threshold	*M* = 4.36, *SD* = 3.85 10 (2.9)
**Birth Type**	
Vaginal Unassisted Vaginal Assisted Elective Caesarean Section Emergency Caesarean Section Missing	201 (54.0) 45 (12.1) 81 (21.8) 44 (11.8) 1 (0.3)
**Gestational Age** (*n* = 369)	*M* = 38.66 weeks, *SD* = 2.16, range: 28–42
**Satisfaction with Child Birth** (*n* = 361)	*M* = 36.79, *SD* = 9.51
**Breastfeeding Status**	
Not Currently Breastfeeding Currently Breastfeeding	34 (9.1) 338 (90.9)
**Perceived Social Support**	
Not enough support Good support Missing	49 (13.2) 321 (86.3) 2 (0.5)
**Relationship Satisfaction** (*n* = 364)	*M* = 4.67, *SD* = 1.39

### Univariate Associations Between MIB and Predictors

As can be seen in Table 6, poorer maternal-infant bonding was associated with higher maternal education status, antenatal and postnatal EPDS, T2 DASS-Depression and DASS-Anxiety, and T2 infant difficult infant temperament.

### Unique and Combined Prediction of MIB from the Putative Predictors

**Antenatal predictors.** All predictors of MIB were examined using hierarchical stepwise linear regression (see Table 4). Poorer MIB at T2 was predicted by the following antenatal (T0) variables, which explained 8.4% variance: younger maternal age, higher maternal education, and higher antenatal depression symptoms (*R* ^2^ = 0.084, adjusted *R* ^2^ = 0.069, *F*(6, 351) = 5.39, *p* < .001). For each one-point change in maternal age, MIB changed by − 0.18. For each one-point change in mothers’ level of education, MIB changed by 1.32. For each one-point change in antenatal depression symptoms, MIB changed by 0.45.

**Table 3:** Description of the Sample from Data Collected at Laboratory Visit when the Child was Aged Between 5-11 Months

	*n* (%)
**Perceived Social Support**	
Not enough support Good support Missing	254 (68.3) 118 (31.7) 0 (0.0)
**Breastfeeding Status**	
Not Currently Breastfeeding Currently Breastfeeding	181 (48.7) 191 (51.3)
**Relationship Satisfaction** (*n* = 371)	*M* = 4.11, *SD* = 1.54
**Mother’s Mental Health During Past Week**	
Depression Subscale (*n* = 372) Anxiety Subscale (*n* = 372)	*M* = 1.35, *SD* = 2.24 *M* = 1.51, *SD* = 2.29
**Difficult Infant Temperament** (*n* = 381)	*M* = 3.90, *SD* = 1.07
**Maternal-Infant Bonding**	*M* = 6.49, *SD* = 6.87

**Table 4:** Stepwise linear regression model predicting maternal-infant bonding at T2 from T0 antenatal predictors (*N* = 357)

	Univariate correlation	*B (SE B)*	β	*t*	R^2^ (R^2^ adj)	ΔR^2^
**Step 1**					0.034 (0.028)**	0.034**
Maternal Age Mothers’ Highest Level of Education Achieved	− 0.07 0.14**	− 0.18 (0.09) 1.18 (0.36)	− 0.119* 0.179***	-2.17 3.28		
**Step 2**					0.082 (0.074)***	0.048***
Maternal Age Mothers’ Highest Level of Education Achieved Antenatal depression	− 0.07 0.14** 0.21***	-0.19 (0.08) 1.26 (0.35) 0.43 (0.10)	− 0.121* 0.191*** 0.220***	-2.28 3.58 4.31		
**Step 3**					0.084 (0.069)***	0.003
Maternal Age Mothers’ Highest Level of Education Achieved Antenatal depression Maternal experiences of child abuse Antenatal perceived social support Attitudes towards pregnancy	− 0.07 0.14** 0.21*** 0.02 − 0.02 0.03	− 0.18 (0.08) 1.32 (0.36) 0.45 (0.11) -0.46 (1.49) 1.63 (2.37) 0.63 (0.87)	− 0.117* 0.200*** 0.227*** − 0.016 0.036 0.038	-2.17 3.67 4.26 -0.31 0.687 0.72		

*Note.* Higher MIB scores indicate poorer bonding *p < .05; **p < .01, ***p < .001

**Table 5:** Stepwise linear regression model predicting maternal-infant bonding at T2 from T1 birth-related predictors (*N *= 333)

	Univariate correlation	*B (SE B)*	β	*t*	R^2^ (R^2^ adj)	ΔR^2^
**Step 1**					0.034 (0.028)**	0.034**
Maternal Age Mothers’ Highest Level of Education Achieved	− 0.07 0.14**	− 0.18 (0.09) 1.18 (0.37)	− 0.119* 0.179**	-2.10 3.28		
**Step 2**					0.107 (0.099)***	0.073***
Maternal Age Mothers’ Highest Level of Education Achieved Postnatal depression	− 0.07 0.14** 0.28***	-0.19 (0.08) 1.02 (0.36) 0.64 (0.12)	− 0.124* 0.154** 0.272***	-2.28 2.83 5.20		
**Step 3**					0.108 (0.092)***	0.002
Maternal Age Mothers’ Highest Level of Education Achieved Postnatal depression Maternal experiences of child abuse Postnatal perceived social support Attitudes towards pregnancy	− 0.07 0.14** 0.28*** 0.02 − 0.06 0.03	-0.19 (0.09) 1.05 (0.37) 0.63 (0.13) -0.35 (1.52) -0.42 (1.48) 0.60 (0.89)	− 0.124* 0.160** 0.269*** − 0.012 − 0.015 0.036	-2.24 2.86 5.03 -0.23 -0.29 0.67		
**Step 4**					0.118 (0.094)***	0.010
Maternal Age Mothers’ Highest Level of Education Achieved Postnatal depression Maternal experiences of child abuse Postnatal perceived social support Attitudes towards pregnancy Satisfaction with childbirth Breastfeeding status Gestation	− 0.07 0.14** 0.28*** 0.02 − 0.06 0.03 0.03 − 0.01 0.06	− 0.20 (0.09) 1.07 (0.37) 0.66 (0.13) -0.01 (1.53) -0.46 (1.48) 0.49 (0.89) 0.07 (0.05) -0.83 (1.46) 0.25 (0.22)	− 0.126* 0.163** 0.281*** 0.000 − 0.016 0.030 0.076 − 0.030 0.058	-2.27 2.89 5.19 -0.01 0.31 0.56 1.42 -0.57 1.11		

*Note.* Higher MIB scores indicate poorer bonding *p < .05; **p < .01, ***p < .001

**Table 6:** Stepwise linear regression model predicting maternal-infant bonding at T2 from T0, T1 and T2 predictors (*N* = 329)

	Univariate correlation	*B (SE B)*	β	*t*	R^2^ (R^2^ adj)	ΔR^2^
**Step 1**					0.070 (0.002)	0.005
Child Age at T2	0.07	0.02 (0.02)	0.070	1.29		
**Step 2**					0.040 (0.032)**	0.035**
Child Age at T2 Maternal Age	0.07 − 0.06	0.02 (0.02) − 0.18 (0.09)	0.081 − 0.117*	1.52 -2.10		
Mothers’ Highest Level of Education Achieved	0.15**	1.22 (0.37)	0.185**	3.31		
**Step 3**					0.160 (0.145)***	0.120***
Child Age at T2 Maternal Age Mothers’ Highest Level of Education Achieved Antenatal depression Postnatal depression Concurrent depression	0.07 − 0.06 0.15** 0.22*** 0.29*** 0.30***	0.02 (0.01) -0.18 (0.08) 1.16 (0.35) 0.16 (0.12) 0.34 (0.14) 0.72 (0.20)	0.06 − 0.115* 0.175** 0.083 0.148* 0.204***	1.24 -2.18 3.31 1.42 2.42 3.59		
**Step 4**					0.187 (0.156)***	0.027
Child Age at T2 Maternal Age Mothers’ Highest Level of Education Achieved Antenatal depression Postnatal depression Concurrent depression Maternal experiences of child abuse Antenatal perceived social support Postnatal perceived social support Concurrent perceived social support Attitudes towards pregnancy Concurrent anxiety	0.07 − 0.06 0.15** 0.22*** 0.29*** 0.30*** 0.02 − 0.05 − 0.07 − 0.15** 0.06 0.26***	0.02 (0.01) -0.19 (0.08) 1.24 (0.35) 0.17 (0.12) 0.35 (0.14) 0.48 (0.26) -1.13 (1.47) 3.52 (2.34) 0.92 (1.45) -2.50 (0.93) 0.53 (0.85) 0.36 (0.25)	0.056 − 0.123* 0.188** 0.086 0.153* 0.136 − 0.039 0.080 0.034 − 0.139** 0.032 0.102	1.11 -2.32 3.51 1.44 2.50 1.85 -0.77 1.51 0.63 -2.69 0.64 1.44		
**Step 5**					0.198 (0.158)***	0.011
Child Age at T2 Maternal Age Mothers’ Highest Level of Education Achieved Antenatal depression Postnatal depression Concurrent depression Maternal experiences of child abuse Antenatal perceived social support Postnatal perceived social support Concurrent perceived social support Attitudes towards pregnancy Concurrent anxiety Satisfaction with childbirth Breastfeeding status at birth Breastfeeding status at T2 Gestation	0.07 − 0.06 0.15** 0.22*** 0.29*** 0.30*** 0.02 − 0.05 − 0.07 − 0.15** 0.06 0.26*** 0.03 − 0.03 0.05 0.07	0.01 (0.01) -0.19 (0.08) 1.26 (0.37) 0.18 (0.12) 0.38 (0.14) 0.44 (0.26) -0.84 (1.48) 3.52 (2.34) 0.85 (1.46) -2.66 (0.95) 0.44 (0.85) 0.38 (0.26) 0.05 (0.05) -0.33 (1.43) -0.19 (0.92) 0.36 (0.20)	0.053 − 0.125* 0.191** 0.090 0.165** 0.123 − 0.029 0.081 0.031 − 0.148** 0.027 0.107 0.055 − 0.012 − 0.011 0.090	1.04 -2.33 3.46 1.50 2.66 1.65 -0.57 1.50 0.58 -2.81 0.52 1.50 1.07 -0.23 -0.21 1.77		
**Step 6**					0.234 (0.193)***	0.036***
Child Age at T2 Maternal Age Mothers’ Highest Level of Education Achieved Antenatal depression Postnatal depression Concurrent depression Maternal experiences of child abuse Antenatal perceived social support Postnatal perceived social support Concurrent perceived social support Attitudes towards pregnancy Concurrent anxiety Satisfaction with childbirth Breastfeeding status at birth Breastfeeding status at T2 Gestation Difficult Infant Temperament	0.07 − 0.06 0.15** 0.22*** 0.29*** 0.30*** 0.02 − 0.05 − 0.07 − 0.15** 0.06 0.26*** 0.03 − 0.03 0.05 0.07 0.31***	0.01 (0.01) -0.17 (0.09) 1.07 (0.36) 0.14 (0.11) 0.30 (0.14) 0.46 (0.26) -0.57 (1.45) 3.58 (2.29) 0.84 (1.43) -2.85 (0.93) 0.10 (0.83) 0.29 (0.25) 0.03 (0.46) -0.44 (1.40) -0.60 (0.908) 0.33 (0.20) 1.62 (0.42)	0.043 − 0.108* 0.162** 0.071 0.130* 0.130 − 0.020 0.082 0.031 − 0.158** 0.005 0.082 0.037 − 0.016 − 0.035 0.081 0.205***	0.86 -2.05 2.98 1.21 2.12 1.78 -0.39 1.56 0.59 -3.07 0.12 1.16 0.73 -0.32 -0.66 1.63 3.90		

*Note.* Higher MIB scores indicate poorer bonding *p < .05; **p < .01, ***p < .001

**Birth-related predictors.** All predictors of MIB were examined using hierarchical stepwise linear regression (see Table 5). Poorer MIB at T2 was predicted by the following birth-related (T1) variables, which explained 11.8% variance: younger maternal age, higher maternal education, and higher postnatal depression symptoms (*R* ^2^ = 0.118, adjusted *R* ^2^ = 0.094, *F*(6, 351) = 5.39, *p* < .001). For each one-point change in mothers’ level of education, MIB changed by 1.07. For each one-point change in postnatal depression symptoms, MIB changed by 0.66.

**All possible predictors.** All predictors of MIB were examined, controlling for infant age at T2, using hierarchical stepwise linear regression (see Table 6). Poorer MIB at T2 was predicted by the following variables, which explained 23.4% variance: younger maternal age, higher maternal education, higher postnatal (T1) depression symptoms, lower concurrent perceived social support (T2), and higher concurrent (T2) difficult infant temperament (*R* ^2^ = 0.234, adjusted *R* ^2^ = 0.193, *F*(17, 323) = 5.80, *p* < .001). For each one-point change in maternal age, MIB changed by − 0.17. For each one-point change in mothers’ level of education, MIB changed by 1.07. For each one-point change in T1 postnatal depression symptoms, MIB changed by 0.30. For each one-point change in T2 concurrent perceived social support, MIB changed by -2.85. For each one-point change in difficult infant temperament, MIB changed by 1.62.

## Discussion

The aim of the current study was to test the role of depression, anxiety, antenatal attitudes towards the pregnancy, perceived social support, maternal history of maltreatment, dissatisfaction with childbirth, breastfeeding, difficult infant temperament, gestation, maternal age and maternal education as unique predictors of poor MIB. As expected, it was found that poorer MIB was associated with higher maternal education level (T0), higher depression symptoms (T0, T1, and T2), anxiety symptoms (T2), and difficult infant temperament (T2), and lower concurrent perceived social support (T2). Contrary to expectations, MIB was not significantly correlated with maternal age, maternal experience of childhood abuse (T0), perceived social support at T0 and T1, attitudes toward pregnancy (T0), satisfaction with childbirth (T1), breastfeeding status (T1 and T2), or the infants’ gestation (T1).

Three models were run to examine the unique longitudinal and concurrent predictors of poor MIB. Regression analyses revealed that poorer MIB when infants were in their first year of life was predicted antenatally by younger maternal age, higher maternal education and higher antenatal depression symptoms. Results showed that maternal experiences of child abuse, attitudes towards pregnancy, and perceived social support did not significantly predict poor MIB above and beyond maternal age, maternal education and antenatal depression symptoms. These results indicate that when mothers are being screened antenatally for risk factors for poor MIB that those mothers who are younger with higher education and higher antenatal depression symptoms should be the primary targets for further screening, and possibly intervention.

Second, a regression model was run with the birth-related (T1) variables predicting poor MIB. Regression analyses revealed that poorer MIB when infants were in their first year of life was predicted at birth by younger maternal age, higher maternal education and higher postnatal depression symptoms. Results showed that models that included maternal experiences of child abuse, perceived social support at birth, attitudes towards pregnancy, breastfeeding status, and gestation did not significantly predict poor MIB over and above younger maternal age, higher maternal education and higher postnatal depression symptoms. Similar to the antenatal results, the postnatal model indicates that when mothers are being screened following the birth of their child for poor MIB risk factors that younger mothers with higher education and higher postnatal depression symptoms should be the primary targets for further assessment, and possibly intervention.

Third, a regression model was run with poor MIB as the outcome variable, and all T0, T1, and T2 variables predicting poor MIB, controlling for the age of the child at the T2 assessment. Regression analyses revealed that poorer MIB when infants were in their first year of life was predicted by younger maternal age, higher maternal education (T0), higher postnatal depression (T2), lower concurrent perceived social support (T2), and higher difficult infant temperament (T2) when controlling for child age at the T2 assessment. As expected, several variables were not significant predictors of poor MIB during the first year of an infant’s life once other predictors were accounted for; specifically: antenatal and concurrent depression (T0 and T2), maternal experiences of child abuse, perceived social support antenatally and at birth (T0 and T1), attitudes towards pregnancy, concurrent anxiety (T2), satisfaction with childbirth, breastfeeding status (T1 and T2), and infant gestation. The current study extends previous research by considering a range of antenatal (i.e. T0), birth (i.e. T1), and postnatal (i.e. T2) factors together within a sample of socio-demographically diverse families who were recruited as part of a birth-cohort study.

These findings support the notion that perinatal mental health is an important factor in the sequelae of poor MIB. Although concurrent anxiety and concurrent depression symptoms were significantly bivariately correlated with poor MIB, they were not significant predictors in the final multivariate regression models. Antenatally, higher depression symptoms were independently predictive of poorer MIB. Similarly, following birth, postnatal depression symptoms were independently predictive of poorer MIB. Thus, these results indicate that mothers who are showing symptoms of depression antenatally or postnatally are at a higher risk of bonding problems within the first year of their child’s life. Interestingly, antenatal and concurrent depression symptoms were not significant predictors in the model with all predictors; however, postnatal depression symptoms remained a significant predictor. It is possible that this may be due to the stability of depression symptoms in this sample (i.e. depression measures were significantly correlated – albeit, they did not appear to show high collinearity). Overall, our maternal depression and poor MIB findings echo those found by various researchers cross-sectionally [5, 10, 16], and longitudinally [11, 12, 72], and indicate the important role of depression in the sequelae of bonding problems.

This study reiterated the importance of examining child characteristics alongside mother characteristics and experiences when examining poor MIB. This study showed that negative infant temperament influenced poor MIB above and beyond all other predictors in the model. Our findings support those of Nolvi et al. [16] who found a significant unique relationship of mother- and father-reported child temperament with poor MIB, over and above maternal depression or anxiety. It is increasingly acknowledged that the infant is not a passive participant in mother-infant interactions [16], and the bidirectional nature of mother-infant relationships is likely reflected in our findings. In particular, our results show that the mother-infant bond is influenced by what the infant brings to the interaction, particularly in terms of his/her temperament.

Concurrent perceived social support was independently associated with poor MIB above and beyond several other factors, with lower levels statistically predicting more bonding problems. This is interesting because it was not a significant independent predictor antenatally at T0 or postnatally at T1, contradicting previous findings that poor satisfaction with social support systems during pregnancy were associated with lower quality mother-infant bonding [25, 26]. When examining our three perceived social support variables, it appears that the majority of mothers reported that they perceived their support networks to be good antenatally and postnatally (i.e. within a month following the birth of their child). At T2 (when their child was 5- to 11-months old), however, more mothers perceived they were not getting enough support, at which timepoint low social support emerged as a significant predictor of poor MIB. It is possible that this change in ratings may be occurring as some mothers’ anticipated social support does not meet their reality, thus creating more variance within this measure. Alternatively, social support may be reducing in the second half of the first year of life for some mothers, which may be impacting their ability to bond with their infant. For example, fathers may have parental leave to provide support in the postnatal days (i.e. T1) and be back to work in these later months (i.e. T2), leaving mothers with less support. Similarly, mothers may have returned to work, in part or full, by T2 and may be juggling work alongside caregiving demands. Regardless of the exact mechanism, this study shows that low perceived social support is linked to poor MIB independent of postnatal depression. Clinicians and researchers, therefore, need to be mindful of *when* in the perinatal period they ask mothers about their social support, and that perceptions of social support may change over time.

In relation to demographic factors, we found that maternal age and education level were significant predictors of poor MIB. Previous research has shown that women with lower levels of education reported better bonding with their infant compared to women with higher education levels [4, 5, 38], and these findings were replicated in this study. It is possible that these findings may relate to the amount of time that the mother spends with her infant. For example, it is possible that when compared to less educated mothers, more educated mothers may be more likely to be working and therefore have less time to bond with their infant. Future research may wish to investigate this possibility further. Moreover, some studies have shown that younger women have reported higher quality bonding with their infants than older women [4, 38], whereas other studies have found no association between poor MIB and maternal age [6, 13, 51]. In the present study, however, these findings were not replicated as younger mothers reported more bonding problems than older mothers. It is unclear why age-related findings are so mixed. Researchers have proposed that social desirability relating to MIB may differ according to age [4, 38]. To our knowledge, only one study has included a measure of social desirability in relation to MIB [5], finding that mothers scoring higher on social desirability also reported stronger MIB than mothers who scored lower on social desirability. It is also possible that older mums may be more likely to be employed in workplaces with good parental leave policies (i.e. that allow for longer leave entitlements than the standard leave provided by the Australian government), affording them more opportunity to bond with their infants. Future research could include a measure of social desirability and length of leave alongside age and MIB to further elucidate these relationships.

Although this study has many strengths, it also has a number of noteworthy limitations. First, this study employed a range of self-report measures, including our measure of MIB. Researchers have emphasized that MIB needs to be self-reported, given that it is measuring self-perceptions [3, 38]; however, future research may employ clinician ratings of predictors including mental health symptomology, and observer/father/other caregiver ratings of difficult infant temperament. As individuals with depression have been theorised and shown to have a negative information processing biases [e.g., 73, 74–76], these filters may bias interpretations of infants’ difficult temperament. By employing an independent rater (either observer or fathers/other caregivers) the shared method variance between mother ratings could be reduced, which in turn would reduce the likelihood that mothers are skewing ratings of infant temperament. Second, T2 measures (including DASS-Depression and Anxiety, T2 perceived social support, and difficult infant temperament) were collected at the same time as the PBQ, so that temporal ordering and causality cannot be determined. Third, in this study, we were unable to examine anxiety separately until the T2 assessment because the antenatal and postnatal data used in this study were routinely collected in hospitals during antenatal midwife visits and postnatal nursing visits. Thus, we were unable to examine any additional predictors of poor MIB, such as antenatal anxiety. It is therefore possible that anxiety could play an earlier role in the development of MIB, but we were unable to examine this in the present study. Fourth, it would have been useful to more broadly investigate difficulties with infants’ sleep and/or feeding (not just whether the mother was breastfeeding at the time of the assessment), as these tend to be major issues present at this age (e.g., [77]). Further, infant sleeping and feeding problems are linked with maternal psychopathology and perceptions of infant temperament [78]; yet have not been extensively investigated with poor MIB. Due to testing load limitations, further information on sleeping and feeding could not be ascertained. Fifth, there was a possible attrition bias from those initially recruited for the larger ‘Watch Me Grow for REAL’ study. Our sample was likely to be older, more educated, to have perceived lower social support, and experienced more postnatal depression symptoms following the birth of their child than the general population. Thus, findings should be interpreted with these limitations in mind.

This study also had several strengths and there are a range of clinical practice implications. A key strength is that this study includes prospective longitudinal data from an ethnically diverse birth-cohort sample. Further, another notable strength is that we were able to consider many variables concurrently within the same statistical model unlike the majority of the MIB literature. To our knowledge, this is also the first birth cohort study to report attitudes towards pregnancy in relation to MIB. It must be noted that there were a small number of mothers who reported that they had an unplanned pregnancy that they were unhappy about. It was shown that attitudes towards pregnancy were not significantly correlated with MIB in this sample. It is possible that this factor may be more pertinent in clinical cohorts that have been referred to specialist services than it is in general population cohorts. In regard to clinical practice implications, practitioners may be able to better support mothers in developing healthy MIB by addressing modifiable factors, such as maternal depression and social support. Two approaches to intervention may be useful to consider: first, the importance of positive MIB to healthy child development and the factors that can impede this relationship could be communicated within universal interventions (such as antenatal classes and postnatal parenting groups); second, targeted interventions for maternal depression in the perinatal period could also address poor MIB. Interventions could include parenting strategies to promote the maternal-infant bond (e.g., increasing maternal sensitivity, and reducing intrusiveness), and teach cognitive strategies to assist mothers to realistically appraise their bonding. Mothers’ cognitions are a reasonable intervention target as they have been shown to influence mothers’ interactions with their infants [11]. Further, interventions addressing poor MIB may also address how to respond to infants with difficult temperaments. Midwives, nurses, obstetricians, and psychologists may be well-placed to have these conversations and to help facilitate the environment to be able to promote the development of bonding between mothers and their infants.

## Summary

Maternal-infant bonding is important for children’s positive development. Poor maternal-infant bonding is a risk factor for negative mother and infant outcomes. Although researchers have examined individual predictors of maternal-infant bonding, studies typically do not examine several concurrent and longitudinal predictors within the same model. This study aimed to evaluate the unique and combined predictive power of cross-sectional and longitudinal predictors of maternal-infant bonding. Participants were 372 pregnant women recruited from an Australian hospital. Data were collected at antenatal appointments (T0), following their child’s birth (T1), and at a laboratory assessment when the child was 5-11-months-old (T2). Overall, this study aligns with previous research on maternal bonding by demonstrating the strong link between poor MIB and maternal depression symptomology. Uniquely, our findings have uncovered that higher maternal education, lower perceived social support (when infants were, on average, 7-months-old), and higher difficult infant temperament also independently contribute to poor MIB within the infant’s first year of life. Moreover, although prior research has linked maternal experiences of child abuse, satisfaction with childbirth, infant gestation, and breastfeeding status to poor MIB, our findings suggest that these factors do not uniquely predict poor MIB during the critical first year of an infant’s life when other factors are considered. To promote positive MIB, global and targeted interventions in the perinatal period would be beneficial, particularly in relation to maternal psychopathology, social support, and difficult infant temperament.

## Data Availability

The data that support the findings of this study are available on reasonable request from the corresponding author, FD. The data are not publicly available participants of this study did not agree for their data to be shared publicly.
